# The optimization of postoperative radiotherapy in de novo stage IV breast cancer: evidence from real-world data to personalize treatment decisions

**DOI:** 10.1038/s41598-023-29888-z

**Published:** 2023-02-18

**Authors:** Minoru Miyashita, Onyinye B. Balogun, Olufunmilayo I. Olopade, Dezheng Huo

**Affiliations:** 1 Section of Hematology and Oncology, Department of Medicine, University of Chicago, 5841 S. Maryland Ave., MC2000, Chicago, IL 60637 USA; 2 Department of Breast and Endocrine Surgical Oncology, Tohoku University Graduate School of Medicine, Sendai, Japan; 3 Department of Radiation Oncology, Weill Cornell Medicine, New York, NY USA; 4 Department of Public Health Sciences, University of Chicago, Chicago, IL USA

**Keywords:** Cancer, Oncology

## Abstract

Prolonged survival of patients with stage IV breast cancer could change the role of radiotherapy for local control of breast primary, but its survival benefit remains unclear. Our aim is to investigate the survival benefit of radiotherapy in de novo stage IV breast cancer. Stage IV breast cancer patients who received breast surgery and have survived 12 months after diagnosis (landmark analysis) were included in the study from 2010 to 2015 of the National Cancer DataBase. Multivariable Cox models and a propensity score matching were used to control for confounding effects. Of 11,850 patients, 3629 (30.6%) underwent postoperative radiotherapy to breast or chest wall and 8221 (69.4%) did not. In multivariable analysis adjusting for multiple prognostic variables, postoperative radiotherapy was significantly associated with better survival (hazard ratio [HR] 0.74, 95% confidence interval [95%CI] 0.69–0.80; *P* < 0.001). Radiotherapy was associated with improved survival in patients with bone (*P* < 0.001) or lung metastasis (*P* = 0.014), but not in patients with liver (*P* = 0.549) or brain metastasis (*P* = 0.407). Radiotherapy was also associated with improved survival in patients with one (*P* < 0.001) or two metastatic sites (*P* = 0.028), but not in patients with three or more metastatic sites (*P* = 0.916). The survival impact of radiotherapy did not differ among subtypes. The results of survival analysis in the propensity score-matched sub-cohort were precisely consistent with those of multivariable analysis. These real-world data show that postoperative radiotherapy might improve overall survival for de novo Stage IV breast cancer with bone or lung metastasis, regardless of subtypes.

## Introduction

The incidence of de novo stage IV breast cancer, which comprises approximately 6% of all diagnosed breast cancers has not declined despite widespread adoption of population screening for early detection^1^. Since most of stage IV disease is considered incurable, prolonging survival time while maintaining quality of life has been the main goal of treatment for decades. Innovations in systemic treatment have dramatically changed the management of patients with stage IV breast cancer leading to significant improvements in long term survival^2,3^. Several retrospective studies suggest that breast surgery might improve survival for stage IV breast cancer^4–7^, but the results of recent randomized controlled trials (RCTs) are inconsistent on survival benefit of surgery^8–12^. In the ECOG-ACRIN E2108 trial, there was no significant difference in overall survival (OS) between de novo stage IV patients treated with systemic therapy alone and systemic therapy plus locoregional treatment (3-year OS rate 67.9% in systemic therapy alone group vs. 68.4% in systemic therapy plus locoregional treatment group, *P* = 0.63)^9^. While surgical resection of a primary tumor is not currently considered the standard procedure to improve survival of stage IV patients, some patients still undergo breast surgery for various reasons including control of tumor bleeding or patients’ preference in actual clinical practice.

The role of local treatment with radiation for patients with Stage IV breast cancer remains unclear. Radiotherapy (RT) has a strong impact not only on locoregional control but also on OS in patients with early breast cancer^13,14^. Several biological mechanisms have been discussed about the survival benefit of locoregional treatment. Irradiation to the tumor site after removing main tumor might lead to a favorable microenvironment including activating anti-tumor immunity^15–17^, suppressing stem cell expansion^18^, and reducing new metastasis dissemination and colonization^19^. Aside from these biological changes, reducing the locoregional recurrence could contribute to the suppression of spread in distant metastatic sites. These underlying mechanisms suggest there may be a survival benefit for use of RT in Stage IV patients but this has not been described well in the literature. Moreover, the roles of surgery and RT may change as we gain a better understanding of breast cancer heterogeneity and integrate molecularly targeted therapies with immune check point inhibitors to extend survival time and improve quality of life for patients with stage IV disease.

Given that there is no RCT on the effect of postoperative RT in de novo stage IV breast cancer patients, observational studies can provide the urgently needed data on this important clinical question. Here, we conducted the largest study to investigate the survival benefit of RT to breast or chest wall after breast surgery in de novo stage IV breast cancer patients using data from the National Cancer Data Base (NCDB).

## Materials and methods

### Data source

Patient data was retrospectively collected in the NCDB, which is a joint project of the Commission on Cancer (CoC) of the American College of Surgeons and the American Cancer Society^20^. This nationwide database collects patient-level data, clinicopathological factors, treatments, extent of disease, and survival outcomes, from roughly 1500 CoC-accredited facilities that represent more than 70% of newly diagnosed cancers in the United States.

### Study population

**Figure 1:**
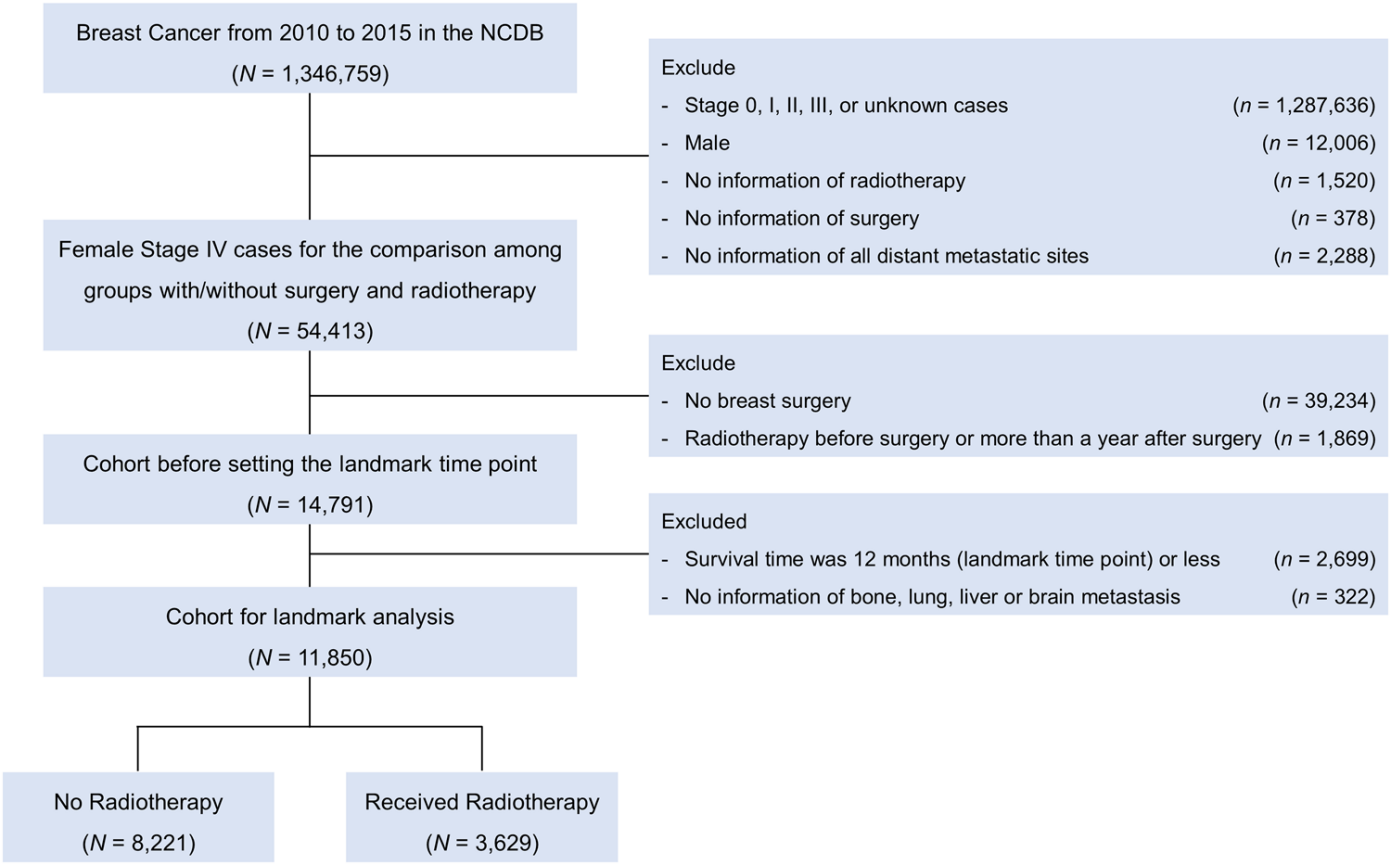
Consort diagram of cohort selection in National Cancer Database (NCDB).

Of 1,346,759 patients diagnosed with breast cancer between January 1, 2010 and December 31, 2015 in the NCDB, we identified 54,413 de novo stage IV female breast cancer for the comparison of characteristics between patients with or without breast surgery and RT after excluding patients without information of RT-targeted sites, surgery and distant metastatic sites (Fig. 1). Then, we selected 14,791 patients who underwent breast surgery. RT administrated to a breast or a chest wall within 12 months of breast surgery was considered as the treatment of interest, and patients who had RT before or more than 12 months after breast surgery were excluded. The exclusion was performed using both the radiation surgery sequence code “Radiation therapy after surgery” and the date from surgery to RT.

To minimize the potential survivor’s bias such that patients who died earlier have no chance to receive RT, we performed conditional landmark analysis. Of 14,791 patients, 4300 patients (29.1%) underwent RT to breast or chest wall after surgery and 10,491 patients (70.9%) did not. Short survival time in de novo metastatic breast cancer may not allow patients and physicians to make a RT choice because disease rapidly progress with high pressure. We set the landmark time to be 12 months because the majority of RT was given within 1 year of diagnosis (Supplemental Fig. 1)^21^. As shown in Supplemental Fig. 2, the proportion of RT was 3.2% among patients who were not alive by 6 months, 17.1% among patients within the 7–12 months survival window, and about 31–38% among patients who survived more than 12 months. The stable treatment membership distribution after 12 months suggests that the choice of 12 months in landmark analysis is reasonable. A total of 2699 patients with survival time less than 12 months were excluded. The patients who received RT after 12 months from diagnosis (landmark time point) remained the ‘No RT group’ to ignore change in group membership after the landmark point. After further excluding patients with missing data on bone, lung, liver or brain metastasis, 11,850 patients were eligible for the survival analysis (Fig. 1).

### Variables and outcomes

The following variables were analyzed as categorical ones; age at diagnosis (cut-off: 55 years), race (white, black, others), the Charlson-Deyo comorbidity index (0, 1, 2 or more) which consists of 15 health conditions^22^. The following clinicopathological factors were analyzed as categorical variables: clinical T stage and clinical N stage according to the American Joint Committee on Cancer (AJCC) Cancer Staging Manual 7th edition, grade (1, 2 or 3, 4), estrogen receptor (ER), progesterone receptor (PgR), human epidermal growth factor receptor 2 (HER2) and breast cancer subtypes (ER positive and/or PgR positive and HER2 negative–Luminal subtype; HER2 positive–HER2 + subtype; or ER negative and PgR negative and HER2 negative–triple negative [TN] subtype). The types of surgery (breast conserving surgery [BCS] or mastectomy), the presence or absence of treatment such as chemotherapy (CT), endocrine therapy (ET) and RT (breast or chest wall) were included as binary variables. Metastatic sites (bone, lung, liver, brain, distant lymph node and others) and the number of metastatic sites (1, 2, 3, 4 or more) were also included as covariates or stratification factors. A survival outcome was measured from date of diagnosis to date of last contact or death as an OS in this study.

### Statistical analysis

Chi-square tests were used to study treatment outcomes between patients who received postoperative RT (breast or chest wall) and those who did not receive postoperative RT. A multivariable Cox proportional hazard model was used to evaluate the independent effects of RT on overall survival, including calculating the adjusted hazard ratios (HR) and 95% confidence intervals (CI). We conducted the association between RT and survival in subgroups, defined by metastatic sites, number of metastatic sites, and molecular subtypes. We also calculated the propensity score of physician's choices of RT using a logistic regression model with RT use as the dependent variable and multiple statistically significant demographic and clinicopathological factors as predictors. Patients were matched by their propensity score ± 0.2 using the nearest-neighbor matching method at a 1:1 ratio between RT group and no RT group. The Kaplan–Meier method with log-rank test was used to estimate survival curves and compare the difference of survival rates in patients between with RT and without RT across metastatic sites, the number of metastatic sites and subtypes. All analyses except for the propensity score matching were performed using SPSS statistical software version 21 (IBM Corporation, Armonk, NY, USA). The propensity score matching was performed using SAS software, JMP Pro 15 (SAS Corp., Cary, NC, USA). All tests were two-sided, and a *P* value of < 0.05 was considered statistically significant.

## Results

### Patient and tumor characteristics

To compare the characteristics among 54,413 de novo stage IV breast cancer patients with or without breast surgery and RT, we found that patients undergoing surgery were more likely to be younger, healthier and have less distant metastasis (Supplemental Table 1). Patient and tumor characteristics in the landmark cohort are shown in Table 1. Of 11,850 patients, 3,629 patients (30.6%) underwent RT to breast or chest wall after surgery (RT group) and 8,221 patients (69.4%) did not (No RT group). Patients with RT are more likely to be younger, have a lower Charlson-Deyo comorbidity index, larger and higher grade tumors, increased lymph node involvement, including ER negative, PgR negative, and HER2 positive hormone receptors. Patients in the RT group were more likely to have undergone mastectomy, chemotherapy and endocrine therapy, and had fewer metastatic sites, compared to patients without RT (Table 1).

**Table 1:** Patient characteristics of 11,850 patients with versus without radiotherapy (RT).

Variables	No RT (N = 8221)		RT (N = 3629)		*P* value
	*N*	%	*N*	%	
Age (years)					< 0.001
Median (Range)	59 (18–90)	55 (22–90)			
< 55	3090	37.6	1781	49.1	
55-	5131	62.4	1848	50.9	
Race					0.191
White	6560	79.8	2855	78.7	
Black	1259	15.3	575	15.8	
Others	347	4.2	177	4.9	
Unknown	55	0.7	22	0.6	
Charlson-Deyo index					< 0.001
0	6772	82.4	3159	87.0	
1	1129	13.7	406	11.2	
2 or more	320	3.9	64	1.8	
cT					< 0.001
is	21	0.3	11	0.3	
1	1369	16.7	428	11.8	
2	2836	34.5	1111	30.6	
3	1211	14.7	619	17.1	
4	1861	22.6	1238	34.1	
Unknown	923	11.2	222	6.1	
cN					< 0.001
0	2676	32.6	702	19.3	
1	2895	35.2	1434	39.5	
2	904	11.0	549	15.1	
3	822	10.0	706	19.5	
Unknown	924	11.2	238	6.6	
Grade					< 0.001
1,2	3550	43.2	1417	39.0	
3,4	3928	47.8	1905	52.5	
Unknown	743	9.0	307	8.5	
ER					< 0.001
Positive	6151	74.8	2577	71.0	
Negative	1922	23.4	1025	28.2	
Unknown	148	1.8	27	0.7	
PgR					0.003
Positive	5056	61.5	2157	59.4	
Negative	2987	36.3	1440	39.7	
Unknown	178	2.2	32	0.9	
HER2					0.020
Positive	2084	25.3	1010	27.8	
Negative	5621	68.4	2452	67.6	
Unknown	516	6.3	167	4.6	
Subtypes					< 0.001
Luminal	4578	55.7	1880	51.8	
HER2 +	2065	25.1	1000	27.6	
Triple-negative	1002	12.2	553	15.2	
unknown	576	7.0	196	5.4	
Breast surgery					< 0.001
BCS	2276	27.7	893	24.6	
Mastectomy	5945	72.3	2736	75.4	
Chemotherapy					< 0.001
Yes	5406	65.8	3154	86.9	
No	2688	32.7	461	12.7	
unknown	127	1.5	14	0.4	
Endocrine therapy					0.020
Yes	4969	60.4	2296	63.3	
No	2965	36.1	1242	34.2	
unknown	287	3.5	91	2.5	
Bone metastasis					< 0.001
Yes	5082	61.8	1897	52.3	
No	3139	38.2	1732	47.7	
Lung metastasis					< 0.001
Yes	1903	23.1	608	16.8	
No	6318	76.9	3021	83.2	
Liver metastasis					< 0.001
Yes	1600	19.5	493	13.6	
No	6621	80.5	3136	86.4	
Brain metastasis					< 0.001
Yes	271	3.3	47	1.3	
No	7950	96.7	3582	98.7	
Distant LN metastasis					< 0.001
Yes	1504	18.3	1022	28.2	
No	5860	71.3	2301	63.4	
unknown	857	10.4	306	8.4	
Other metastasis					0.007
Yes	506	6.2	277	7.6	
No	6858	83.4	3046	83.9	
unknown	857	10.4	306	8.4	
No. of metastatic sites		0.0			< 0.001
1	6110	74.3	3032	83.5	
2	1653	20.1	513	14.1	
3	388	4.7	79	2.2	
4 or more	70	0.9	14	0.4	

*RT* radiotherapy,* cT* clinical T,* cN* clinical,* ER* estrogen receptor, *PgR* progesterone receptor,* HER2* human epidermal growth factor receptor 2,* BCS* breast conserving surgery, *LN* lymph node.

### Univariate and multivariable survival analysis

In the entire cohort for the conditional landmark analysis, 5,421 patients (45.7%) died during the median follow-up time of 34.73 months (range from 12.02 to 95.24 months). The univariate analysis showed that almost all variables except for distant lymph node metastasis (*P* = 0.431) and other metastasis (*P* = 0.229) were significantly associated with OS (Supplemental Table 2). In the multivariable analysis, postoperative RT to breast or chest wall was significantly associated with lower risk of death (HR 0.74, 95% CI 0.69–0.80; *P* < 0.001) (Table 2). Strong prognostic factors for poor survival (all *P* < 0.001) include older age, higher Charlson-Deyo comorbidity index, advanced clinical T stage (cT4), higher tumor grade, and triple-negative subtype (versus luminal subtype). HER2 + subtype was associated with favorable survival compared to luminal subtype (*P* < 0.001). The use of chemotherapy or endocrine therapy was also associated with favorable survival (*P* < 0.001). The presence of metastases in major four metastatic sites, namely bone, lung, liver and brain, were significantly associated with worse prognosis in the multivariable analysis, while higher number of distant metastatic sites was also a prognostic factor. Clinical N stage, race and the type of surgery were not significantly associated with survival outcome in the multivariable analysis (Table 2).

**Table 2:** Multivariable analysis of conditional landmark cohort (N = 8,496).

Variables	*N*	Events	HR	95%CI (lower–upper)		*P* value
All patients	8496	3820				
Radiotherapy						
No	5709	2804	1.00			
Yes	2787	1016	0.74	0.69	0.80	< 0.001
Age						
< 55	3585	1421	1.00			
55-	4911	2399	1.26	1.18	1.35	< 0.001
Race						
White	6790	3028	1.00			
Black	1342	661	1.07	0.99	1.17	0.103
Others	364	131	0.94	0.79	1.13	0.516
Charlson-Deyo score						
0	7119	3064	1.00			
1	1089	584	1.29	1.18	1.41	< 0.001
2 or more	288	172	1.45	1.24	1.70	< 0.001
cT						
1	1400	575	1.00			
2	3181	1382	1.07	0.96	1.18	0.214
3	1487	664	1.15	1.02	1.29	0.021
4	2428	1199	1.28	1.15	1.43	< 0.001
cN						
0	2626	1142	1.00			
1	3479	1525	1.03	0.95	1.12	0.484
2	1157	585	1.13	1.02	1.25	0.025
3	1234	568	1.04	0.94	1.17	0.439
Grade						
1, 2	3829	1463	1.00			
3, 4	4667	2357	1.55	1.44	1.66	< 0.001
Subtypes						
Luminal	4897	2209	1.00			
HER2 +	2389	828	0.61	0.56	0.67	< 0.001
Triple-negative	1210	783	1.68	1.50	1.89	< 0.001
Breast surgery						
BCS	2254	968	1.00			
Mastectomy	6242	2852	1.07	0.99	1.16	0.069
Chemotherapy						
No	2215	1125	1.00			
Yes	6281	2695	0.84	0.77	0.91	< 0.001
Endocrine therapy						
No	3084	1562	1.00			
Yes	5412	2258	0.78	0.72	0.86	< 0.001
Bone metastasis						
Yes	4939	2255	1.00			
No	3557	1565	0.80	0.73	0.87	< 0.001
Lung metastasis						
Yes	1832	968	1.00			
No	6664	2852	0.84	0.76	0.92	< 0.001
Liver metastasis						
Yes	1495	778	1.00			
No	7001	3042	0.71	0.64	0.79	< 0.001
Brain metastasis						
Yes	209	125	1.00			
No	8287	3695	0.70	0.58	0.85	< 0.001
No. of metastatic sites						
1	6554	2736	1.00			
2	1549	840	1.18	1.07	1.30	< 0.001
3 or more	393	244	1.25	1.03	1.50	0.021

*HR* hazard ratio, *CI* confidential interval, *cT* clinical T, *cN* clinical N, *BCS* breast conserving surgery.

**Figure 2:**
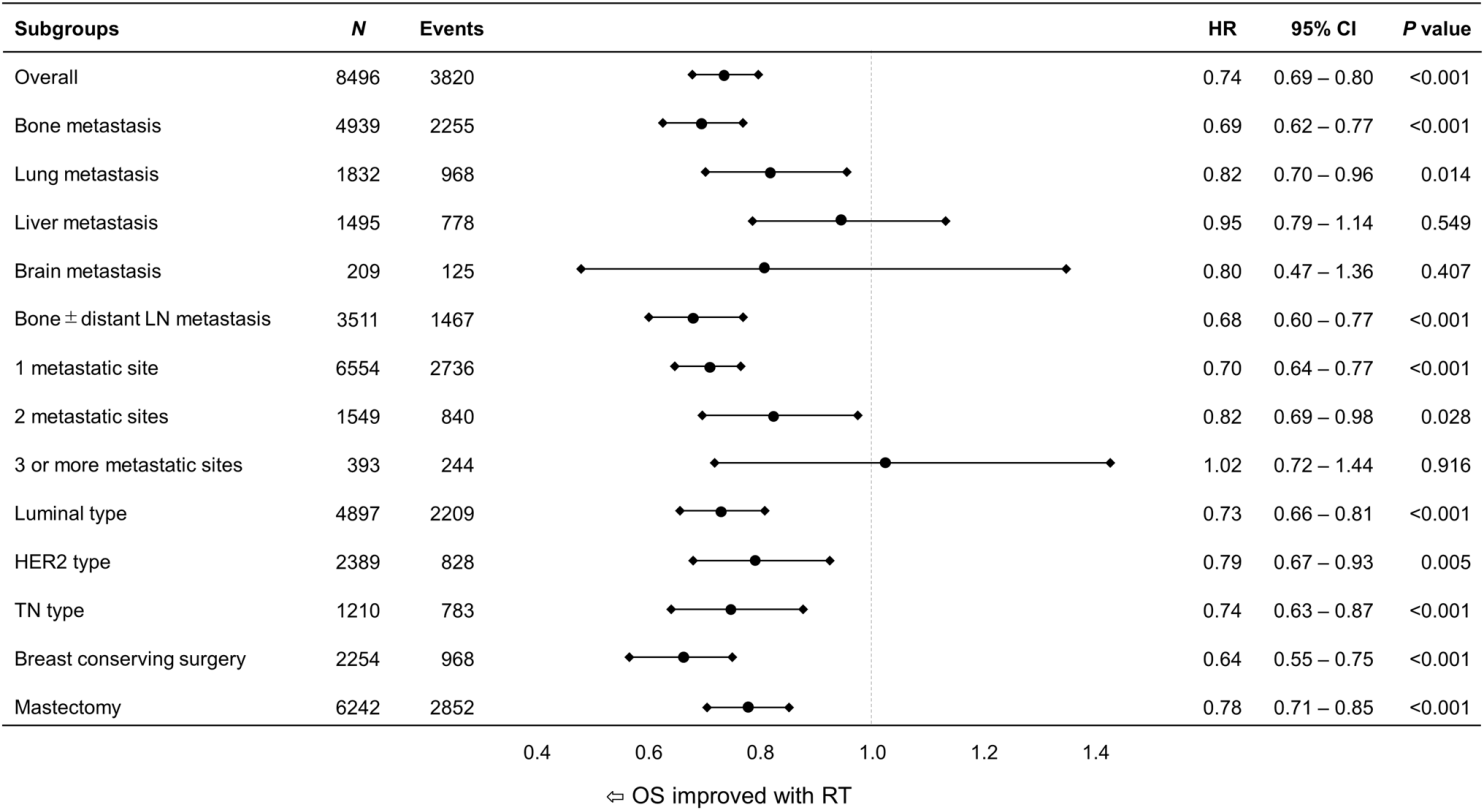
The forest plots showed the hazard ratio (HR) and 95% confidential interval (CI) of radiotherapy in selected subgroups after adjusting for prognostic variables (age, race, Charlson-Deyo comorbidity index, clinical T stage, clinical N stage, grade, subtype, surgery type, chemotherapy, endocrine therapy, bone, lung, liver, brain metastasis and the number of metastatic sites) in multivariable Cox models. *LN* lymph node, *HER2* human epidermal growth factor receptor 2, *TN* triple negative.

According to the clinically meaningful subgroups of the metastatic sites, the number of metastatic sites and the subtypes, we evaluated the association between overall survival and RT after adjusting all of the prognostic variables (age, race, Charlson-Deyo comorbidity index, cT, cN, grade, subtype, surgery type, chemotherapy and endocrine therapy). The forest plots showed that RT was associated with improved survival in patients with bone metastasis (HR 0.69, 95% CI 0.62–0.77; *P* < 0.001) or lung metastasis (HR 0.82, 95% CI 0.70–0.96; *P* = 0.014) as well as patients having only bone with or without distant lymph node metastasis (HR 0.68, 95% CI 0.60–0.77; *P* < 0.001) (Fig. 2). In contrast, no statistically significant association for RT was observed in patients with liver metastasis (HR 0.95, 95% CI 0.79–1.14; *P* = 0.549) or brain metastasis (HR 0.80, 95% CI 0.47–1.36; *P* = 0.407), but few patients had liver or brain metastasis. Patients with bone or lung metastasis have generally more favorable prognosis than patients with liver or brain metastasis. The result indicates that RT has more impact on survival for patients with more favorable metastatic sites. Interestingly, the strength of the association between RT and survival was monotonically related to the number of metastatic sites: HR = 0.70 (95% CI 0.65–0.77; *P* < 0.001) in patients with 1 metastatic site, HR = 0.82 (95% CI 0.69–0.98; *P* = 0.028) in patients with 2 metastatic sites, and HR = 1.02 (95% CI 0.72–1.44; *P* = 0.916) in patients with 3 or more metastatic sites. The association between survival and RT was significant in all three molecular subtypes. When analyzing the impact of RT on OS according to the type of surgery, RT was significantly associated with improved survival in patients receiving BCS or mastectomy with HR = 0.64 (95% CI 0.55–0.75; *P* < 0.001) or HR = 0.78 (95% CI 0.71–0.85; *P* < 0.001), respectively (Fig. 2).

### Propensity score matching and survival analysis

**Figure 3:**
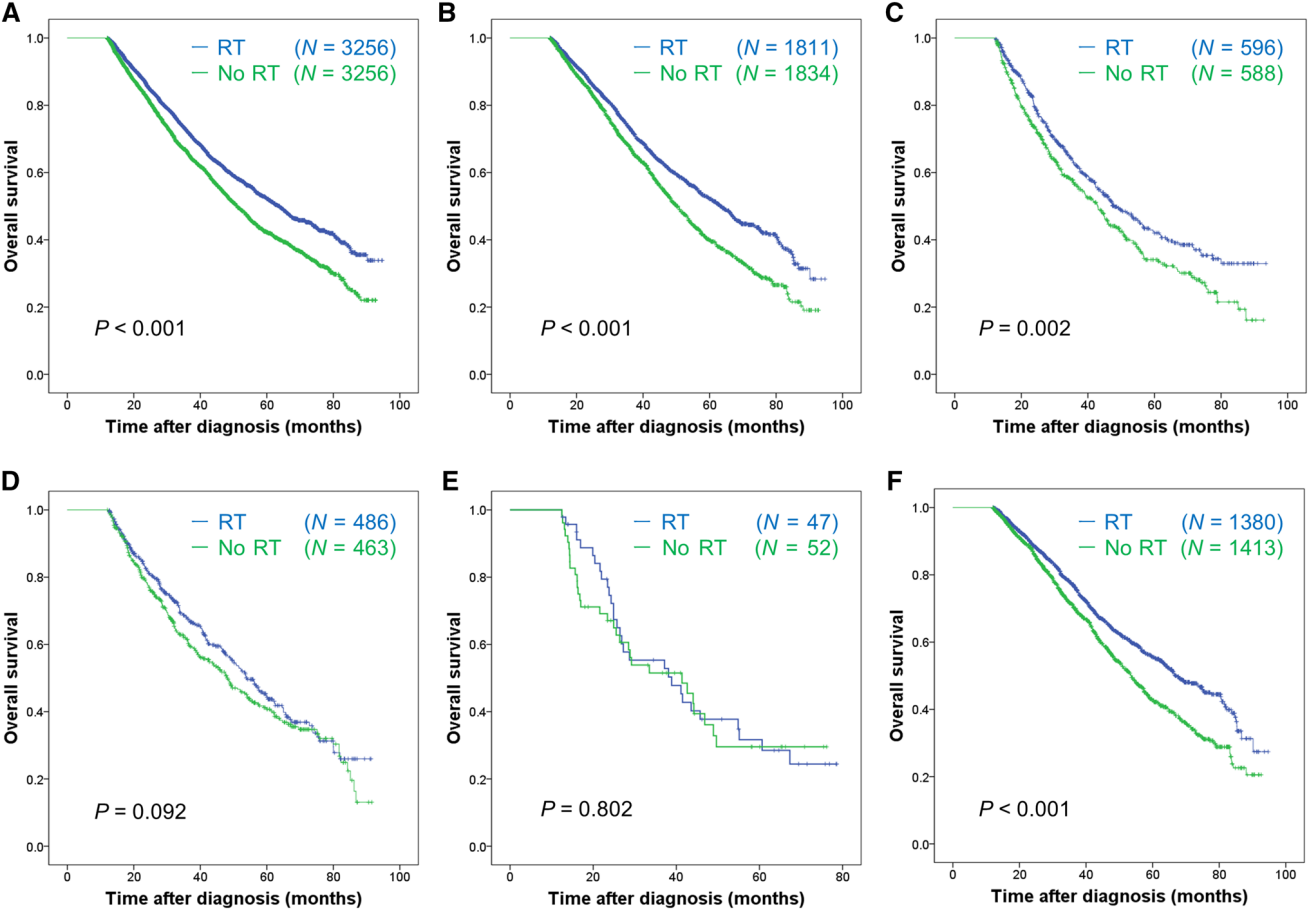
Overall survival of patients with or without radiotherapy in the overall propensity score matched cohort (**A**), the subgroups of bone metastasis (**B**), lung metastasis (**C**), liver metastasis (**D**), brain metastasis (**E**), and bone + /− distant lymph node metastasis (**F**). Log-rank test was used to test the significance of survival difference and *P* values were shown in the figure. In Cox regression models, radiotherapy was significantly associated with improved survival in the overall cohort (HR 0.76, 95% CI 0.71–0.82; *P* < 0.001) (**A**), with bone metastasis (HR 0.74, 95% CI 0.67–0.82; *P* < 0.001) (**B**), lung metastasis (HR 0.77, 95% CI 0.66–0.91; *P* = 0.002) (**C**) or bone + /− distant lymph node metastasis (HR 0.73, 95% CI 0.65–0.82; *P* < 0.001) (**F**), but not in patients with liver metastasis (HR 0.85, 95% CI 0.71–1.01; *P* = 0.093) (**D**) or brain metastasis (HR 0.94, 95% CI 0.57–1.55; *P* = 0.803) (**E**). HR, hazard ratio; CI, confidence intervals.

**Figure 4:**
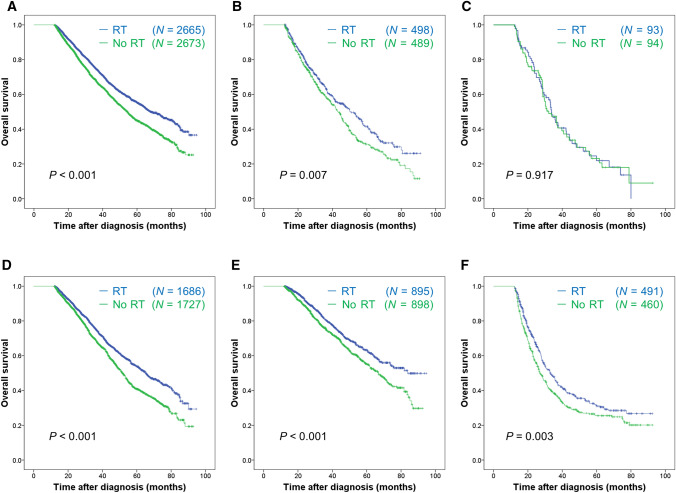
Overall survival of patients with or without radiotherapy in the subgroups of 1 metastatic site (**A**), 2 metastatic sites (**B**), 3 or more metastatic sites (**C**), Luminal subtype (**D**), HER2 + subtype (**E**), and triple-negative subtype (**F**) in the overall propensity score matched cohort. Log-rank test was used to test the significance of survival difference and *P* values were shown in the figure. In Cox regression models, radiotherapy was significantly associated with improved survival in patients with 1 metastatic site (HR 0.75, 95% CI 0.68–0.81; *P* < 0.001) (**A**), or 2 metastatic sites (HR 0.79, 95% CI 0.66–0.94; *P* = 0.007) (**B**), but not in patients with 3 or more metastatic sites (HR 0.98, 95% CI 0.68–1.41; *P* = 0.981) (**C**). Radiotherapy was also significantly associated with improved survival in patients with Luminal subtype (HR 0.74, 95% CI 0.66–0.82; *P* < 0.001) (**D**), HER2 + subtype (HR 0.74, 95% CI 0.63–0.88; *P* < 0.001) (**E**) and triple-negative subtype (HR 0.78, 95% CI 0.66–0.92; *P* = 0.003) (**F**). HR, hazard ratio; CI, confidence intervals.

Propensity score (PS) matching was applied for the landmark cohort to adjust the biased variables between the RT group and the no RT group. 3,256 patients without RT were successfully matched to 3,256 patients with RT; after PS matching, the two groups were well-balanced (Supplemental Table 3). In the matched sub-cohort, 2755 patients died during a median follow-up time of 34.99 months (range 12.02–94.69 months). The results of survival analysis in the propensity score matched cohort were completely consistent with that of multivariable analysis in the entire cohort. Patients receiving postoperative RT to breast or chest wall had a better survival compared to patients without RT (HR 0.76, 95% CI 0.71–0.82; *P* < 0.001) (Fig. 3a). In the subgroup analysis of each metastatic site, RT was associated with improved survival in patients with bone metastasis (HR 0.74, 95% CI 0.67–0.82; *P* < 0.001), lung metastasis (HR 0.77, 95% CI 0.66–0.91; *P* = 0.002), or only bone with/without distant lymph node metastasis (HR 0.73, 95% CI 0.65–0.82; *P* < 0.001), but not in patients with liver metastasis (HR 0.85, 95% CI 0.71–1.01; *P* = 0.093) or brain metastasis (HR 0.94, 95% CI 0.57–1.55; *P* = 0.803) (Fig. 3b–f). RT was associated with improved survival in patients with one metastatic site (HR 0.75, 95% CI 0.68–0.81; *P* < 0.001) or two metastatic sites (HR 0.79, 95% CI 0.66–0.94; *P* = 0.007), but not in patients with three or more metastatic sites (HR 0.98, 95% CI 0.68–1.41; *P* = 0.981) (Fig. 4a–c). RT was associated with better survival regardless of breast cancer subtypes (Fig. 4d–f).

## Discussion

To our knowledge, this is the largest study evaluating the effect of postoperative RT and survival in de novo stage IV breast cancer patients. Using granular information of metastatic sites and the number of them, breast cancer subtypes and treatments from NCDB, we conducted multivariable analysis in conditional landmark cohort as well as propensity score matched analysis to minimize the selection bias between groups as much as possible. We found that postoperative RT was associated with improved survivals in patients with one (hazard ratio 0.70) or two metastatic sites (hazard ratio 0.82), but not in patients with three or more metastatic sites. Regarding de novo Stage IV patients with bone metastasis only, our findings were consistent with the report from a multi-center prospective registry study (BOMET MF 14–01)^23^. The results identified a population of Stage IV patients likely to receive the benefit, including increased survival rates, while also providing physicians and patients with real-world data to improve medical decision-making.

There have been four previous studies which retrospectively evaluated the efficacy of RT after surgery in de novo stage IV breast cancer patients^24–27^. A single institutional study in France with 581 patients showed that locoregional radiotherapy was associated with improved survival^27^. Using data from 2,207 patients from the Surveillance, Epidemiology, and End Results (SEER) database, Kim YJ, et al. concluded that postoperative RT significantly improved survival rates compared with surgery alone especially in patients with synchronous bone metastasis^26^. However, specific information including treatment sites of RT (radiation field) are not available in the SEER database, and as described in their report, the inability to identify whether RT targeted the breast tumor bed or other organs involved as metastases could limit the interpretation of the reported results. Another study using the SEER database has faced the same limitation^24^. Out of 54,413 stage IV female patients studied in the NCDB 2010–2015, 6169 (11.3%) patients received breast or chest wall irradiation and 11,627 (21.4%) patients received RT targeting other organs outside the primary site (the data is not shown in this study). To eliminate this critical contamination, we included only the patients receiving postoperative RT to the breast or chest wall in our study. We also constructed the study cohort for conditional landmark analysis as shorter survival time does not allow some patients to receive RT. In a sensitivity analysis, we found that including patients with survival time less than 12 months overestimated the effect of RT (data not shown). Including this population of shorter survival in previous studies might have led to overstated recommendations about benefit^24–26^. Our study addresses those limitations by providing detailed characteristics of de novo stage IV patients with or without surgery and RT in the real world.

The survival benefit of RT has been proven in early breast cancer^13,14,28,29^. This study provides further evidence about potential benefit in a subset of de novo stage IV patients. On account of adequate control after novel effective systemic therapies, patients with prolonged survival time may further benefit from the addition of RT for loco-regional control of the breast primary. Unfortunately, the underlying mechanism of how RT brings about survival benefits has not been clearly elucidated even though some potential mechanisms are proposed^15–19^. Biological mechanisms related to RT efficacy could be observed regardless of tumor stages. The abscopal effect that RT leads to regression of tumor at distant metastatic sites that are not irradiated^15–19^ might be the reason why RT improved the survival of patients with low distant disease burden across subtypes in our study. Furthermore, the shapes of the Kaplan–Meier curves indicate that the survival advantages of RT became wider over time in patients with bone, lung, or only bone with/without distant lymph node metastasis as well as patients with 1 or 2 metastatic sites. Of a separate note, locoregional treatment had an OS benefit in the present TN subtype cohort (Fig. 4) whereas most of previous reports showed no benefit from locoregional treatment in TN patients. Of 1555 TN patients in the study, 1214 (78%) patients have one metastatic site, 275 (18%) patients have two metastatic sites and 66 (4%) patients have three or more metastatic sites. We can suggest that patients in our study cohort tend to have lower number of distant metastatic site compared to the cohorts of previous reports and this would be the potential reason why locoregional treatment showed an OS benefit in the present TN cohort. When considering insufficient evidence of RT efficacy in stage IV patients, our findings suggest that RT may be considered for these subgroups to achieve the appropriate decision-making. Because there is a physiological and financial burden involved for those patients treated with RT, additional investigational approaches are required in order to ensure the greatest benefit versus risk for those receiving this treatment.

Regarding to the indication of RT in actual clinical practice, physicians might consider RT more for the patients who responded very well to initial systemic therapy and had stabilization of their distant metastasis quickly. Although there was no information of response to initial systemic therapy in NCDB, we performed the additional analysis regarding to the sequence of systemic therapy and surgery. The median duration from diagnosis to surgery is 46 days. 45% of patients received surgery before systemic therapy and 43% of patients received surgery within 2 months after systemic therapy started. The remaining patients received surgery 2 or more months after systemic therapy started. Namely, most of patients were treated with surgery before efficacy of systemic treatment was evaluated. We compared the time duration from systemic therapy to surgery between RT and No RT group among patients who initially received systemic therapy (chemotherapy, antibody drug, hormone therapy and immunotherapy) before surgery. As a result, the median time from systemic therapy to surgery in RT and No RT group is 155 days and 173 days, respectively with no significant difference. Furthermore, the duration from surgery to RT might be better to be discussed because the longtime of period between surgery to RT might associate with RT for locoregional progression or persistent status of metastasis after first treatments. When exploring the days from surgery to RT, 77% of patients received RT within 3 months after surgery and 90% of patients received RT within 6 months after surgery. Therefore, there is a high possibility that almost all patients received so-called ‘planned adjuvant RT’ (Supplemental Fig. 3). The result might support the similarity of two groups in terms of the therapeutic efficacy of initial systemic therapy to distant metastasis. The ECOG-ACRIN E2108 trial reported the favorable locoregional control by locoregional therapy even though patients who did not respond to systemic therapy were excluded^9^. It is unfortunate that there is no information regarding the other endpoints including locoregional recurrence in NCDB.

In our study, several limitations exist. Even though we thoroughly made an effort to minimize confounding between groups in observational studies, we could not control for unmeasured biases such as performance status and the detailed regimens of chemotherapy. Multicollinearity may exist so we examine the correlation among all variables used in the multivariable regressions (Supplemental Table 4). Several factors have influenced each other, e.g. subtype and endocrine therapy, but none of the covariates are strong correlated with radiotherapy, the main variable of interest, so multicollinearity has less impact on the main conclusion. Only future randomized clinical trials can eliminate these unmeasured confounding bias. Our results were generated based on the detailed information in terms of the metastatic site and the number of distant metastases. However, even if there are two or more metastatic sites in the other organs except bone, lung, liver, brain and distant LN metastasis, it has been recorded as one metastatic site or ‘the other metastasis’ in NCDB. Bone, lung, liver, brain and distant lymph node are the main metastatic sites for breast cancer, so the situation that a patient has two or more metastatic sites except the five main sites (bone, lung, liver, brain and distant LN) would be quite rare. This occurred in 4.1% patients in the no RT group and 4.8% in RT group, suggesting that the issue related to the number of metastatic sites except the five main sites was small. The landmark analysis makes the study findings only applicable to patients who have survived 12 months after diagnosis, and should not be generalizable to patients who have survival time < 12 months. The patient background of the cohort might affect the result of multivariable analysis. Some kind of information including direct intervention to metastatic sites, regimen and duration of chemotherapy was not available in NCDB. And the use of endocrine therapy was evaluated in the entire cohort including ER negative tumor as well as the other variables. Regarding of chemotherapy or endocrine therapy, the result needs to be carefully interpreted.

## Conclusion

The results imply that RT to breast or chest wall after surgery might improve survival of de novo stage IV breast cancer patients with bone or lung metastasis who have two or less metastatic sites, except for liver and brain regardless of breast cancer subtypes. Our findings likely have plausible biological mechanisms related to RT that are beyond the scope of this study. Nonetheless, this is the largest study to date and further advances the use of real-world data for medical decision-making about postoperative RT in patients with Stage IV breast cancer who have survived 12 or more months after diagnosis.

## Supplementary Information

Supplementary Information 1.

Supplementary Information 2.

## Data Availability

The data that support the findings of this study are available from the National Cancer Database but restrictions apply to the availability of these data, which were used under data use agreement for the current study, and so are not publicly available. Data are however available from the American College of Surgeons and the Commission on Cancer upon directly data application.
