# Supplementary material for: The optimization of postoperative radiotherapy in de novo stage IV breast cancer: evidence from real-world data to personalize treatment decisions
Source: Sci Rep. 2023 Feb 18;13:2880. doi: 10.1038/s41598-023-29888-z (PMC9938892; doi:10.1038/s41598-023-29888-z)
Supplement: Supplementary file 1 — Supplementary Information 1. [file 41598_2023_29888_MOESM1_ESM.pptx]

## Slide 1
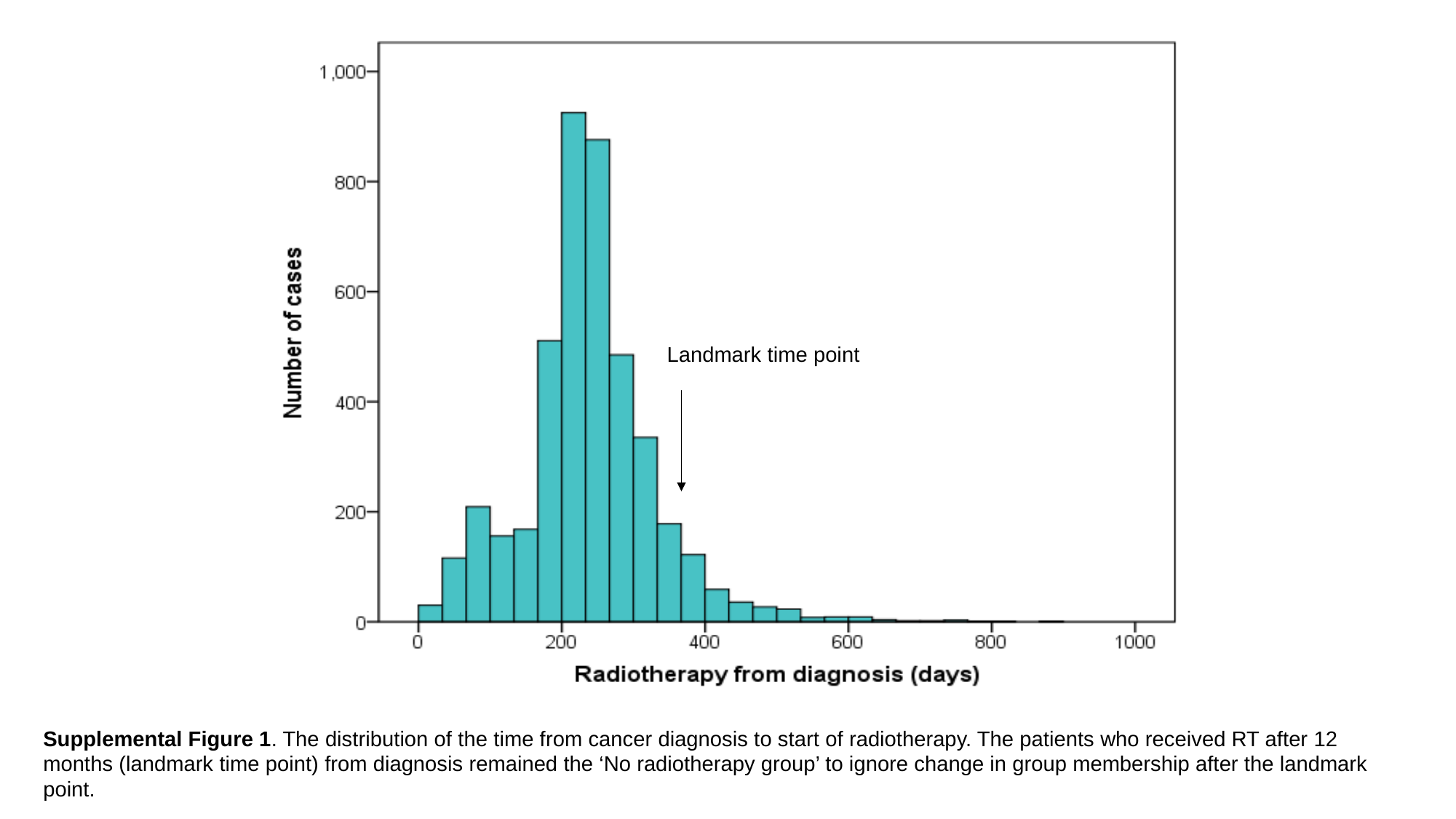

Landmark time point
Supplemental Figure 1. The distribution of the time from cancer diagnosis to start of radiotherapy. The patients who received RT after 12 months (landmark time point) from diagnosis remained the ‘No radiotherapy group’ to ignore change in group membership after the landmark point.

## Slide 2
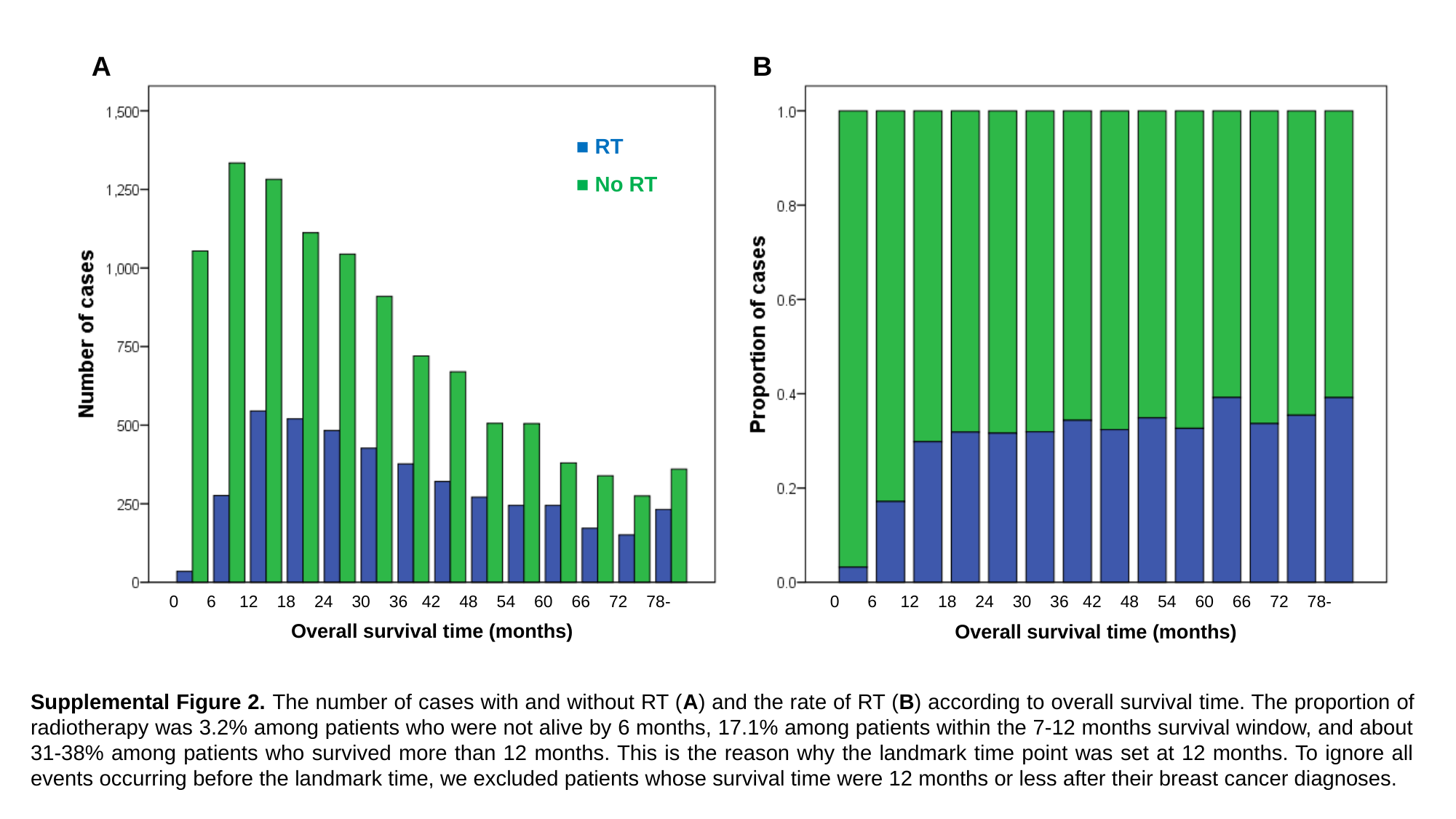

A
B
■ RT
■ No RT
0 6 12 18 24 30 36 42 48 54 60 66 72 78-
0 6 12 18 24 30 36 42 48 54 60 66 72 78-
Overall survival time (months)
Overall survival time (months)
Supplemental Figure 2. The number of cases with and without RT (A) and the rate of RT (B) according to overall survival time. The proportion of radiotherapy was 3.2% among patients who were not alive by 6 months, 17.1% among patients within the 7-12 months survival window, and about 31-38% among patients who survived more than 12 months. This is the reason why the landmark time point was set at 12 months. To ignore all events occurring before the landmark time, we excluded patients whose survival time were 12 months or less after their breast cancer diagnoses.

## Slide 3
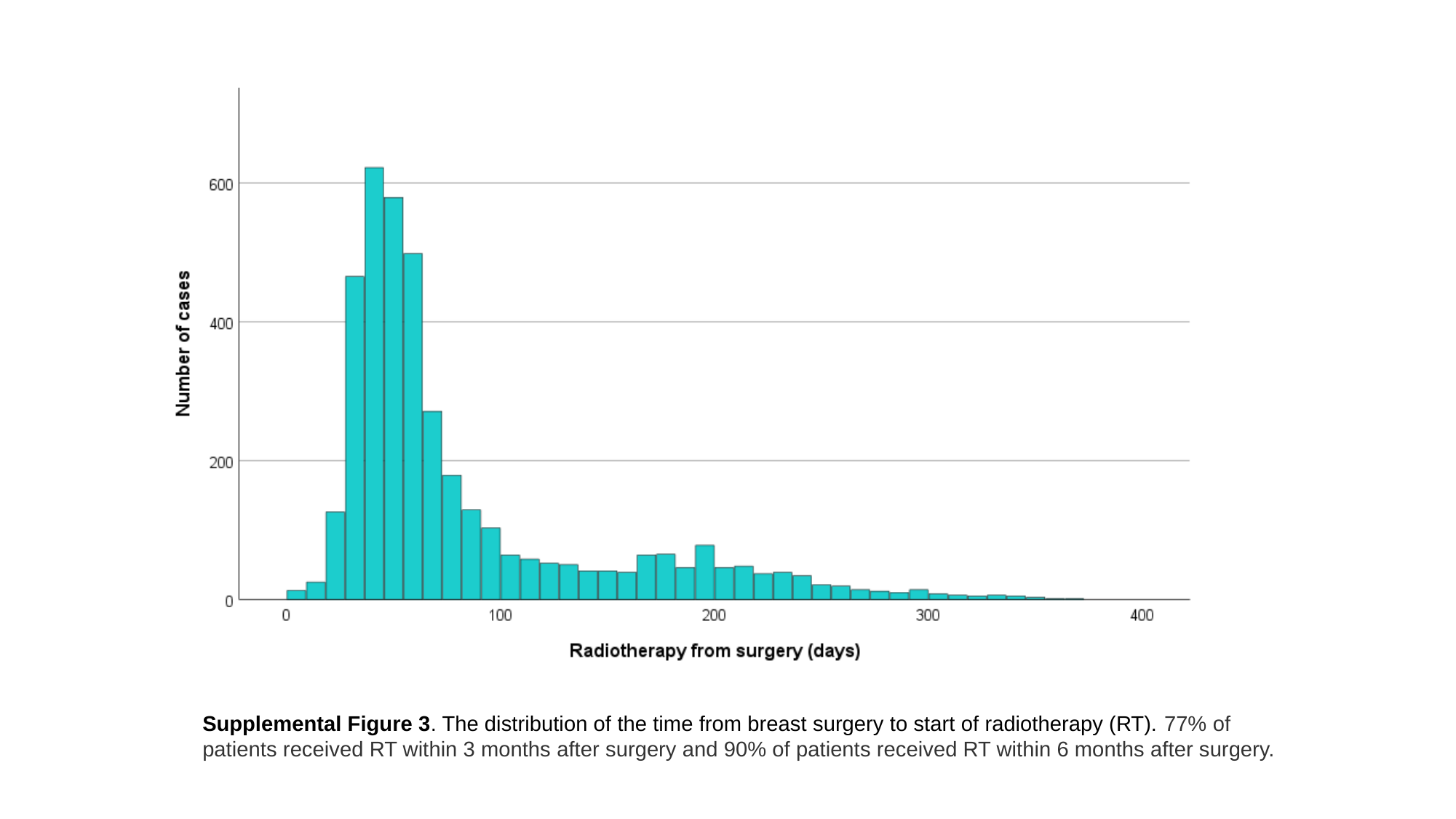

Supplemental Figure 3. The distribution of the time from breast surgery to start of radiotherapy (RT). 77% of patients received RT within 3 months after surgery and 90% of patients received RT within 6 months after surgery.
